# Novel long non‐coding RNA CYB561‐5 promotes aerobic glycolysis and tumorigenesis by interacting with basigin in non‐small cell lung cancer

**DOI:** 10.1111/jcmm.17057

**Published:** 2022-01-22

**Authors:** Longfei Li, Zhimin Li, Jingming Qu, Xiangju Wei, Feng Suo, Jilei Xu, Xiucheng Liu, Chang Chen, Shiying Zheng

**Affiliations:** ^1^ Department of Thoracic Surgery The First Affiliated Hospital of Soochow University Suzhou China; ^2^ Department of Thoracic Surgery Xuzhou Cancer Hospital Xuzhou China; ^3^ Department of Thoracic Surgery Shanghai Pulmonary Hospital Tongji University School of Medicine Shanghai China

**Keywords:** long non‐coding RNA, metabolism, metastasis, non‐small cell lung cancer

## Abstract

Abnormally expressed long non‐coding RNAs (lncRNAs) have been recognized as potential diagnostic biomarkers or therapeutic targets in non‐small cell lung cancer (NSCLC). The role of the novel lnc‐CYB561‐5 in NSCLC and its specific biological activity remain unknown. In this study, lncRNAs highly expressed in NSCLC tissue samples compared with paired adjacent normal tissue samples and atypical adenomatous hyperplasia were identified by RNA‐seq analysis. Lnc‐CYB561‐5 is highly expressed in human NSCLC and is associated with a poor prognosis in lung adenocarcinoma. In vivo, downregulation of lnc‐CYB561‐5 significantly decreases tumour growth and metastasis. In vitro, lnc‐CYB561‐5 knockdown treatment inhibits cell migration, invasion and proliferation ability, as well as glycolysis rates. In addition, RNA pulldown and RNA immunoprecipitation (RIP) assays show that basigin (Bsg) protein interacts with lnc‐CYB561‐5. Overall, this study demonstrates that lnc‐CYB561‐5 is an oncogene in NSCLC, which is involved in the regulation of cell proliferation and metastasis. Lnc‐CYB561‐5 interacts with Bsg to promote the expression of Hk2 and Pfk1 and further lead to metabolic reprogramming of NSCLC cells.

## 1 INTRODUCTION

Lung cancer is the second most prevalent type of cancer and the leading cause of cancer deaths worldwide. Epidemiological studies show that the incidence is on the rise globally and more than 80% of all new lung cancer cases are non‐small cell lung cancer (NSCLC).^1^, ^2^ Despite the groundbreaking advances the understanding of the role of the immune system in the control and treatment of lung cancer, it remains the leading cause of cancer death worldwide.^3^

Long non‐coding RNAs (lncRNAs) are defined as a group of ncRNA transcripts of more than 200 nucleotides in length with no or limited protein‐coding capability.^4^ Current literature generally agrees that lncRNAs directly participates in the initiation and the development of various human diseases by affecting epigenetic mechanisms, interfering with transcription or regulating biological processes.^5^, ^6^, ^7^ Over the last decade, numerous studies have revealed that lncRNAs participate in the progress of various types of tumours.^8^, ^9^, ^10^ LnRNAs can regulate the proliferation, metastasis and metabolic program of tumour cells, showing tumour‐suppressive or ‐promoting (oncogenic) functions.^11^, ^12^, ^13^ In addition, tumour‐associated chronic inflammation is a hallmark of cancer that fosters progression to a metastatic stage, and lncRNAs may interfere with inflammation in tumoral microenvironment thus promote tumour growth.^14^, ^15^ Recently, Yu and co‐workers identified 64 lncRNAs significantly dysregulated in 57 NSCLC tumour samples compared with 16 normal tissues and determined that multiple lncRNAs were associated with tumorigenesis and histological differentiation.^16^ However, the specific biological activity of many lncRNAs that are closely related to tumorigenesis and their specific biological activities remains unknown. Therefore, conducting more research to elucidate the detailed mechanism of action of lncRNAs will have far‐reaching significance for improving lung cancer diagnosis and treatment strategies.

In this study, we identified a novel lncRNA, namely lnc‐CYB561‐5 (Gene ID: ENSG00000233635), which is markedly upregulated in malignant lung cancer and potentially correlated with the prognosis of NSCLC patients in The Cancer Genone Atlas (TCGA) database. Further studies suggested that the high expression of lnc‐CYB561‐5 in lung cancer cells significantly promotes cell proliferation, migration and invasion in vitro and in vivo. Therefore, we propose that lnc‐CYB561‐5 may be closely involved in the pathogenesis and progression of NSCLC and has the potential to be used as a biomarker of poor prognosis.

The overall purposes of this study are as follows: (1) to investigate the role of lnc‐CYB561‐5 in NSCLC. (2) to elucidate the detailed mechanism of action of lnc‐CYB561‐5 in regulating the initiation and progression of NSCLC.

## 2 MATERIAL AND METHODS

### 2.1 Animals

BALB/c nude mice (weighing about 16 g, at 6–8 weeks of age) were purchased from Shanghai Model Organisms (Shanghai, China). Mice were housed 5 mice per cage, in a controlled environment (at room temperature, 22–25°C; humidity, 50%–60%; under a 12‐h light/dark cycle), and provided *ad libitum* access to food and water. In this study, all experiments were performed in adherence with the National Institutes of Health (NIH Publication, 8th Edition, 2011; NIH, Bethesda, MD, USA) guidelines on the use of laboratory animals. The care and experimental protocols for laboratory animals were approved by the Animal Care and Use Committee of Xuzhou Medical University (201806W008).

### 2.2 Clinical samples

A series of human NSCLC tissue samples were obtained from the pathology department of Xuzhou Cancer Hospital. All specimens were pathologically confirmed as NSCLC, and the patients had not received radiotherapy or chemotherapy before surgery. After resection, the tumour and adjacent tissues were frozen in liquid nitrogen, and the specimens were immediately stored at −80°C. The specimens were collected under the guidance of the U.S. Health Insurance Portability and Accountability Act (HIPAA) protocol and supervised by the ethics committee of the hospital.

### 2.3 RNA sequencing assay and data analysis

For human NSCLC tissue samples, high‐throughput RNA sequencing analysis was performed by Shanghai Aksomics Biotech Company Ltd. Briefly, total RNA was extracted from each sample and the concentration and quality of the total RNA were determined using a NanoDrop ND‐1000 spectrophotometer (Thermo Fisher Scientific Inc.). The ribosomal RNA (rRNA) was removed from the total RNA using the NEBNext^®^ rRNA Depletion Kit (New England Biolabs, Inc.) following the manufacturer's instructions. RNA libraries were constructed by using NEBNext^®^ Ultra™ II Directional RNA Library Prep Kit (New England Biolabs, Inc.) according to the manufacturer's instructions. The high‐quality reads were aligned against the human reference genome (UCSC HG19), guided by the Ensembl gtf gene annotation file, with the hisat2 software (v2.0.4). Then, HTSeq software (v0.9.1) was used to obtain the transcript level (lncRNA) raw count as the expression profiling, and edgeR (v3.16.5) was used to perform normalization, and differentially expressed mRNAs were identified by p‐value and fold change.

Similarly, total RNAs from the H1299 cells treated with sh‐lnc‐CYB561‐5 treatment and control cells were isolated and quantified. Gene Ontology (GO) term enrichment analysis and Kyoto Encyclopedia of Genes and Genomes (KEGG) pathway enrichment analysis were performed based on the top 50 differentially expressed mRNAs.

### 2.4 Cell culture and treatment

The human cancer cell lines (H1299 (Cat No. FH0908), A549 (Cat No. FH0045), H292 (Cat No. FH0085) obtained from FuHeng Biology (Shanghai, China); SW900 (Cat No. BNCC275987) obtained from BNBIO (Beijing, China); PC9 (Cat No. MZ‐2682) obtained from Mingzhou Bio), were cultured as previously reported.^17^, ^18^ The human bronchial epithelial cell lines (HBE; BNBIO, Cat No. BNCC338600) and BEAS‐2b (FuHeng Biology, Cat No. FH0319) were cultured in Dulbecco's modified eagle medium (DMEM) purchased from HyClone Laboratories Inc. supplemented with 5% foetal bovine serum and 1% penicillin/streptomycin solution, at 37°C in a humidified atmosphere containing 5% CO_2_.

### 2.5 Preparation of plasmids, lentivirus and stable cells

Short hairpin RNAs (shRNAs) were designed and synthesized by GenePharma. The shRNA‐ctrl vector and shRNA‐lnc‐CYB561‐5 were transfected into H1299 and H292 cells using Lipofectamine 2000 transfection reagent (Invitrogen). Recombinant lentivirus (Bsg‐LV, Vector‐LV) were prepared by Genechem Co., Ltd. The titre of the concentrated virus suspension was 5 × 10^11^ Tu/L. Cells were infected with lentivirus for 48 h and then selected with 2 ng/ml puromycin for 2 weeks, with the medium refreshed every 3 days.

### 2.6 Reverse transcription‐PCR and qRT‐PCR

TRIzol reagent was used to extract the total RNA from human lung tissue and cell lines. The cDNA was generated with random primers using the Reverse Transcription System (Promega Corporation). Beta‐actin was used for the normalization of the qRT‐PCR data. Primer sequences used in this study are listed in Table S1.

### 2.7 Cell proliferation, invasion and migration assays

Cell proliferation ability was determined using a Cell Counting Kit‐8 (CCK‐8; KeyGen Biotech) and a colony formation assay as previously reported.^19^ Briefly, 5 × 10^3^ cells were seeded in 96‐well plates, and recommended volume of CCK‐8 solution was added at 24, 48, 72 and 96 h, respectively. For the colony formation assay, 1 × 10^3^ cells were cultured in 6‐well plates at 37°C for 10 days. The visible colonies were fixed with 4% paraformaldehyde and stained with crystal violet. The area of colony formation was measured using ImagePro Plus 6.0 software (Media Cybernetics, Silver Spring).

The migration and invasion assays were performed as previously described.^19^ For the migration assay, 4 × 10^4^ H1299 cells or H292 cells were seeded in serum‐free medium in the upper chamber and incubated for 24 h at 37℃. Cells that traversed the membrane were stained with crystal violet (0.04%) and counted under a microscope. For the invasion assay, the Transwell filter inserts were coated with Matrigel (Cat No.356237; 1:10; BD Biosciences), and 1 × 10^5^ H1299 cells or H292 cells were seeded in serum‐free medium in the upper chamber and incubated for 48 h at 37℃.

### 2.8 Analysis of apoptosis by flow cytometry

The Annexin V fluorescein isothiocyanate (FITC)/propidium iodide (PI) apoptosis detection kit (KeyGen Biotech) was used for the detection of apoptotic cells with corresponding treatment. Briefly, 0.5 ml of binding buffer was added into 1 × 10^5^ cells. Sequentially, the cells were stained with PI at room temperature for 15 min and then analysed by flow cytometry on a FACSCanto^™^ II flow cytometer (BD Biosciences).

### 2.9 Western blotting analysis

For Western blot analysis, an equal amount of protein was separated on a 12.5% sodium dodecyl sulphate‐polyacrylamide gel electrophoresis (SDS‐PAGE) pre‐cast gel (KeyGen Biotech Co., Ltd.) and electro‐transferred to 8 μm nitrocellulose membranes (Millipore). After blocking with 5% non‐fat milk for 1 h at room temperature, the samples were incubated with the corresponding primary antibody against Pfk1 (Affinity, Cat No. DF7362; 1:1000), G6pi (Affinity, Cat No. DF13660; 1:500), HK1 (Proteintech, Cat No. 15656–1‐AP), HK2 (Proteintech, Cat No. 66974–1‐Ig), Eno1 (Affinity, Cat No. DF2920; 1:1000), Glut1 (CST, Cat No. 12939), Bsg (Proteintech, Cat No. 11989–1‐AP), Lbr (Proteintech, Cat No. 12398–1‐AP) and β‐catenin (Proteintech, Cat No. 51067–2‐AP, 1:1000) at 4°C for 12 h. Then, the membranes were incubated with the corresponding fluorescently labelled secondary antibody, including anti‐mouse or anti‐rabbit antibodies (Li‐Cor Biosciences) for 2 h at room temperature. Eventually, signals were detected by Odyssey Infrared Imaging System (Li‐Cor Biosciences). Digitized images were analysed using the ImageJ software (NIH).

### 2.10 Extracellular flux measurements

The extracellular acidification rate (ECAR) and oxygen consumption rate (OCR) were measured by the Seahorse XFe96 Analyzer (Seahorse Bioscience/Agilent Technologies). Glycolytic rates were measured with the Seahorse XF glycolytic rate assay (S7805A, Seahorse Agilent).

### 2.11 TCGA data analysis

This study was performed using TCGA publication guidelines. The clinical data and expression level of lnc‐CYB561‐5 in NSCLC (including 533 cases with lung adenocarcinoma (LUAD) and 502 cases of lung squamous carcinoma (LUSC)) and normal tissue were downloaded and extracted from the TCGA database.

### 2.12 Gene co‐expression network analysis

The gene co‐expression network was performed as previously reported.^20^ Briefly, the Pearson correlation coefficients between the expression profiles of lnc‐CYB561‐5 and differentially expressed mRNA were calculated to determine the co‐expression relationships of lnc‐CYB561‐5 and mRNA expression. The protein‐coding genes (PCGs) with (Pearson correlation coefficients > 0.8, *p *< 0.05, FDR < 1) were treated as the potential lncRNAs‐related mRNA.

### 2.13 RNA pulldown assay

RNA pulldown assays were performed as previously described.^21^ Briefly, biotin‐labelled RNAs (antisense RNA) were transcribed using Biotin RNA Labeling Mix (Promega Corporation) and T7 RNA polymerase treated with RNase inhibitor and purified with a Clean‐up kit (Promega Corporation). The biotinylated lnc‐CYB561‐5 probes were dissolved in binding and washing buffer and incubated with streptavidin agarose resin (Thermo Fisher Scientific Inc.). Then, H1299 cell lysates were incubated with probe‐coated streptavidin beads, and the pulled‐down proteins were run on SDS‐PAGE gels. Samples were prepared for Western blotting analysis, as described above.

### 2.14 RNA immunoprecipitation

The RIP assay was performed using the EZ‐Magna RIP Kit (Millipore) according to the manufacturer's protocol using 5 mg of antibody. H1299 cells were lysed in complete RIP lysis buffer, and the cell extract was incubated with protein A/g agarose beads conjugated with the antibody against Bsg or control IgG for 2 h at 4°C. Beads were washed and incubated with Proteinase K to remove proteins. Eventually, the purified RNA was subjected to qRT‐PCR analysis.

### 2.15 Statistical analysis

In this study, all of the data are expressed as the means ± SD and were processed using the SPSS 20.0 software (IBM Corporation). Multiple group comparisons were performed using one‐way analysis of variance (ANOVA) test followed by the least significant difference t test for post hoc analysis. Data between two independent groups were compared using a two‐tailed Student *t*‐test. *p* < 0.05 was considered as a significant difference.

## 3 RESULTS

### 3.1 **Lnc‐CYB561‐5 is upregulated and associated with a poor prognosis in NSCLC**.

**FIGURE 1:**
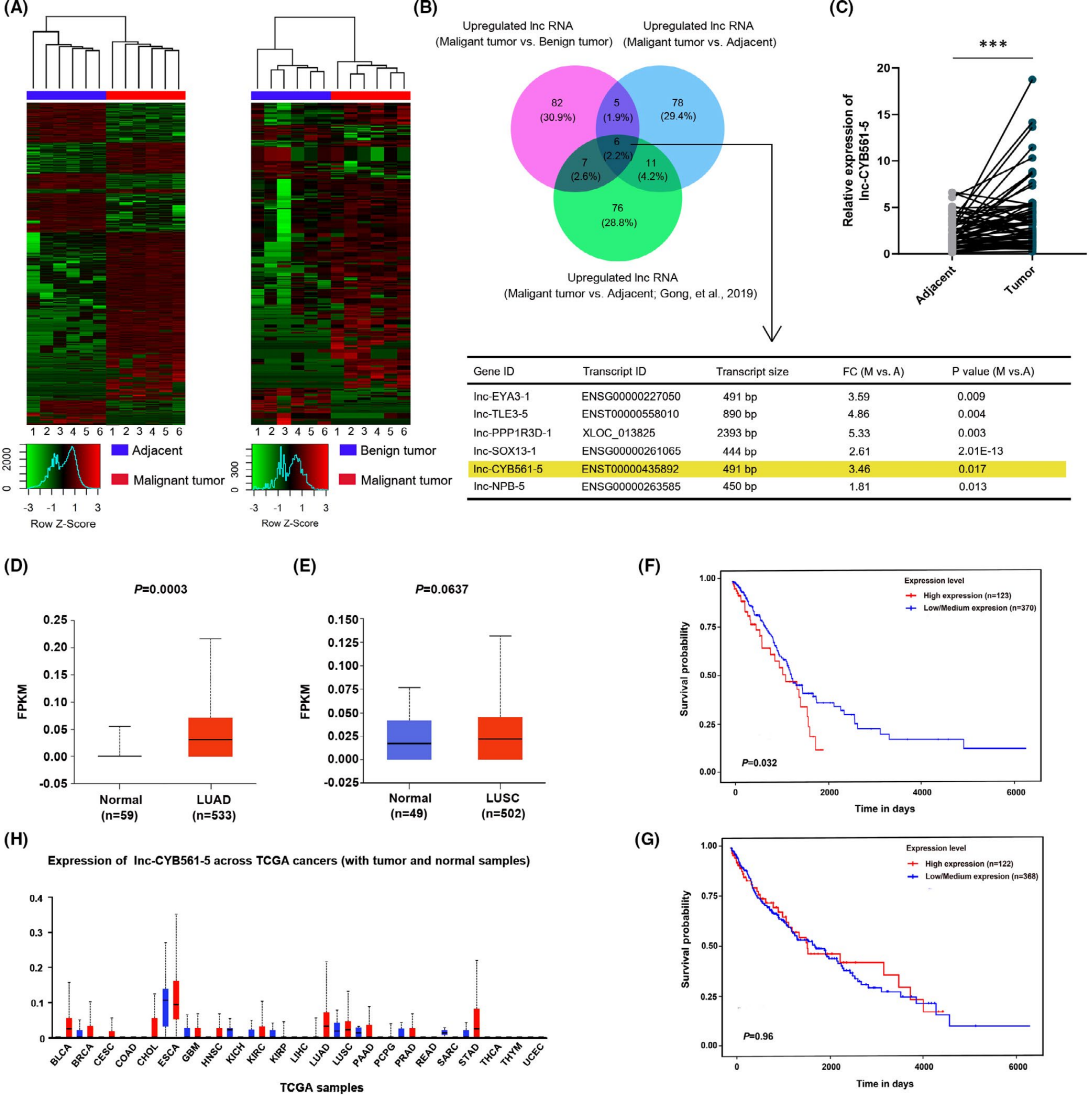
Lnc‐CYB561‐5 is upregulated in NSCLC and is associated with a poor prognosis. (A) Hierarchical clustering showing differentially expressed lncRNAs in NSCLC tissues compared with that in tumour‐adjacent tissue (Left); differentially expressed lncRNAs in NSCLC tissues compared with that in atypical adenomatous hyperplasia (Right). (B) Venn diagram showing 6 commonly upregulated lncRNAs. (C) Detection of lnc‐CYB561‐5 expression in NSCLC tissues and paracancerous tissues by qRT‐PCR, ***p < 0.001, *n* = 74. (D) The expression of lnc‐CYB561‐5 in LUAD tissues and normal tissues was analysed according to TCGA database. (E) The expression of lnc‐CYB561‐5 in LUSC tissues and normal tissues was analysed according to the TCGA database. (F and G) Kaplan‐Meier survival curve analyses were performed to explore the effects of lnc‐CYB561‐5 on the survival rate in LUAD and LUSC. (H) The overall lnc‐CYB561‐5 expression in multiple human cancers from TCGA

The hierarchical clustering in Figure 1A shows differentially expressed lncRNAs in 6 NSCLC tissues and tumour‐adjacent tissue and 6 atypical adenomatous hyperplasia (fold change <0.5 or >2, and *p* < 0.05). Among the top 100 genes (ranked by *p* value and fold change), 11 lncRNAs were significantly upregulated in NSCLC compared with benign tumours and tumour‐adjacent tissue. Furthermore, a comparison of the upregulated lnc RNAs with the data from the study by Gong et al.^22^ and the result indicated that 6 lncRNAs, namely lnc‐EYA3‐1, lnc‐TLE3‐5, lnc‐PPP1R3D‐1, lnc‐SOX13‐1, lnc‐CYB561‐5 and lnc‐NPB‐5, were both upregulated in NSCLC tissues (Figure 1B).

In addition, using PCR primers designed for lnc‐CYB561‐5, we confirmed by qRT‐PCR analysis that lnc‐CYB561‐5 was significantly upregulated in both LUAD and LUSC tissues by qRT‐PCR analysis (*p *< 0.05) (Figure 1C, Figure S1A). However, analysis of TCGA data sets showed that lnc‐CYB561‐5 was only significantly expressed in LUAD, but did not appear to play a significant role in biological functions of LUSC. The discrepancy between our data and TCGA database may be due to the different ethnicity of the samples investigated. Correlation analysis demonstrated that high levels of lnc‐CYB561‐5 were associated with tumour stage (*p *< 0.05) and lymph node metastasis (*p *< 0.05). However, there was no significant relationship between lnc‐CYB561‐5 expression and other factors, including age (*p *> 0.05) and sex (*p *> 0.05) (Supplementary Table S, Figure S1B and Figure S1C). The data from TCGA database showed that the expression of lnc‐CYB561‐5 in LUAD tissues was upregulated and reduced survival, but there was no difference in the level of lnc‐CYB561‐5 between LUSC and adjacent tissues (Figure 1D–G). In addition, the results of the expression analysis of lnc‐CYB561‐5 across TCGA cancers (tumour versus normal samples) was shown in Figure 1H, revealed that lnc‐CYB561‐5 was highly expressed in most malignant tumours, including bladder urothelial carcinoma, breast invasive carcinoma, cholangiocarcinoma and stomach adenocarcinoma.

### 3.2 **Lnc‐CYB561‐5 facilitates NSCLC cell proliferation in vitro and in vivo**.

**FIGURE 2:**
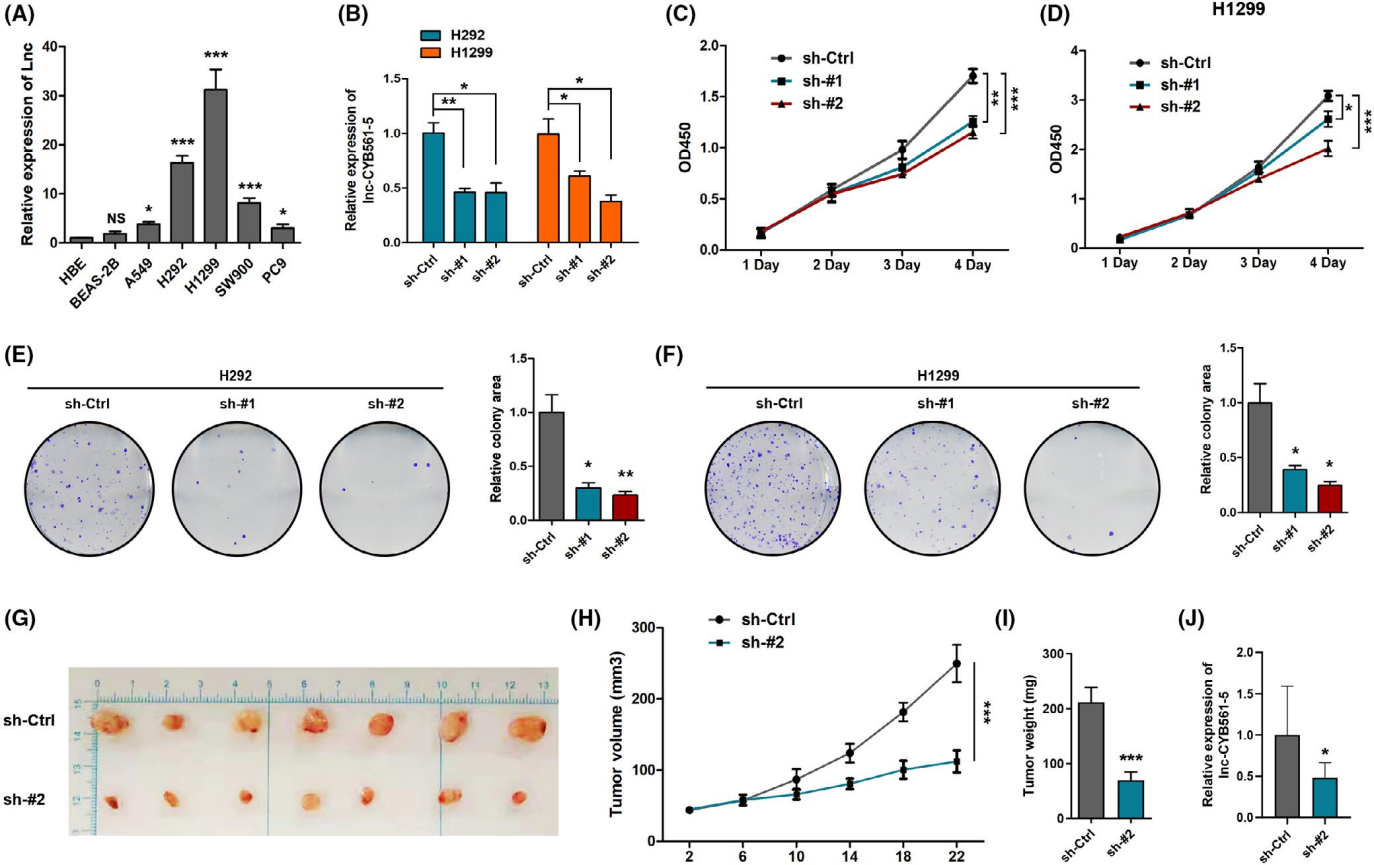
Lnc‐CYB561‐5 facilitates NSCLC cell proliferation in vitro. (A) The expression level of lnc‐CYB561‐5 was detected by qRT‐PCR in HBE, BEAS‐2B and NSCLC cell lines (A549, H1299, H292, SW900 and PC9), *p < 0.05, ***p < 0.001, NS, *p *> 0.05 vs the HBE cells. (B) Knockdown of lnc‐CYB561‐5 was confirmed in H1299 and H292 cells by qRT‐PCR, *p < 0.05, **p < 0.01 vs the indicated group. (C and D) CCK‐8 assay analysis of cell proliferation in H1299 and H292 cells, *p < 0.05, **p < 0.01, ***p < 0.001 vs. the indicated group. (E and F) Colony formation assays in H1299 and H292 cells, *p < 0.05, **p < 0.01 vs. the sh‐Ctrl group, *n* = 5. (G–I) Photographs of matrigel plugs excised from mice after 22 days of growth in vivo and quantitative analysis of the tumour volume and tumour weight, ***p < 0.001 vs. the sh‐Ctrl group, *n* = 7. (J) Detection of lnc‐CYB561‐5 expression in xenograft models by qRT‐PCR, *p < 0.05, *n* = 3. Data are presented as means ± SEM. Multiple group comparisons were performed using one‐way ANOVA followed by Tukey's post hoc test. Two‐group comparisons were performed using unpaired *t*‐test

To elucidate the biological functions of lnc‐CYB561‐5 in NSCLC cells, the expression levels of lnc‐CYB561‐5 in HBE cells, BEAS‐2B cells and a series of lung cancer cell lines, including A549, SW900, H1299, H292 and PC9 cells, were measured by qRT‐PCR analysis. The expressions of lnc‐CYB561‐5 showed great heterogeneity among lung cancer cell lines (Figure 2A). We also investigated the effect of lentivirus‐mediated shRNA knockdown targeting lnc‐CYB561‐5 in H1299 and H292 cells, which express the highest level of lnc‐CYB561‐5 (Figure 2B). The CCK‐8 and colony formation assays revealed that knockdown of lnc‐CYB561‐5 significantly inhibited the growth and proliferation of H1299 and H292 cells (Figure 2C–F), while lnc‐CYB561‐5 overexpression treatment was found to promote the cell proliferation in A549 cells (Figure S2A). The role of lnc‐CYB‐561–5 in the proliferation of lung cancer cells was further explored in mice xenograft models established by subcutaneous injection of sh‐lnc‐CYB561‐5‐#2 treated H1299 cells (Figure 2G). The result indicated that a low level of lnc‐CYB561‐5 significantly reduced the tumour quality and tumour volume (*p *< 0.05) (Figure 2H–J).

### 3.3 **The effects of lnc‐CYB‐561‐5 on lung cancer cell apoptosis and metastasis**.

**FIGURE 3:**
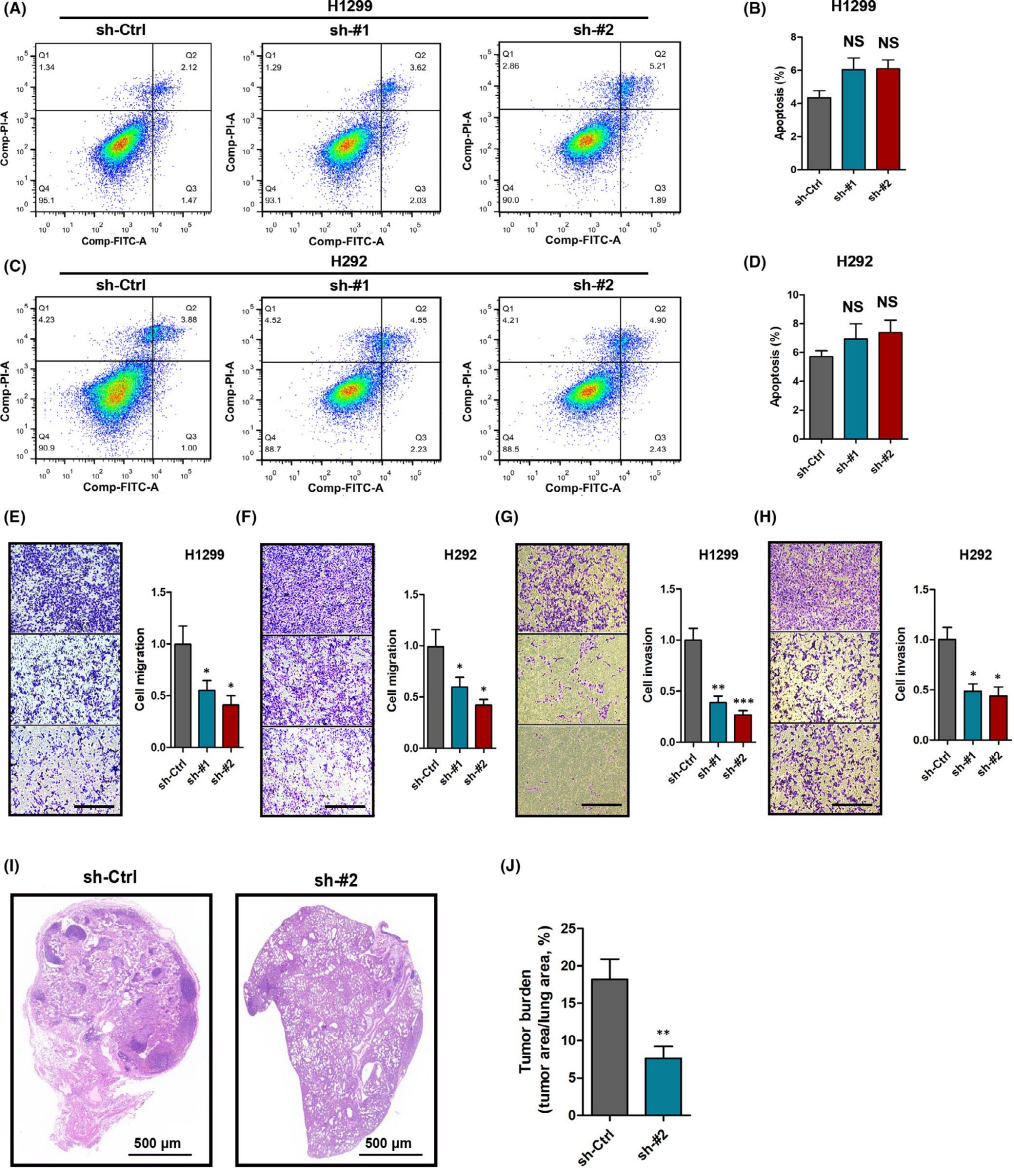
Effects of lnc‐CYB‐561–5 on lung cancer cell apoptosis and metastasis. (A–D) Flow cytometric analysis of cells apoptosis in H1299 and H292 cells. (E–H) The migration and invasion of H1299 cells and H292 cells with sh‐lnc‐CYB561‐5 treatment, bar = 100 μm. (I) Representative lung sections of in vivo metastasis assays, bar = 500 μm. (J) Quantitative statistics of tumour burden. *p < 0.05, **p < 0.01, ***p < 0.001, NS, *p *> 0.05 vs. the sh‐Ctrl group, *n *= 5. Data are presented as means ± SEM. Multiple group comparisons were performed using one‐way ANOVA followed by Tukey's post hoc test. Two‐group comparisons were performed using unpaired *t*‐test

Flow cytometric analysis revealed that there was no significant difference in the level of apoptosis between the control group and the lnc‐CYB561‐5 knockdown group in H1299 and H292 cells (Figure 3A–D). Hongbin J et al.^23^ identified adenocarcinoma metastatic program genes upregulated in A549 and H2126 cells (lung cancer). In this study, the mRNA expressions of CD24, Fgb, Glrx, Map7 and Vimentin were found to be highly upregulated in lnc‐CYB561‐5‐overexpressing A549 cells (Figure S2B). We subsequently performed transwell assays to determine the effects of lnc‐CYB561‐5 on the migration and invasion of H1299, H292 and A549 cells, and the results showed that a low level of lnc‐CYB561‐5 was closely associated with the invasion ability of lung cancer cell (Figure 3E–H, Supplementary Figure 2C). In in vivo metastasis assays, lnc‐CYB561‐5 knockdown also inhibited metastasis of H1299 cells to the lungs after tail vein injection (Figure 3I, J).

### 3.4 Lnc‐CYB561‐5 promotes aerobic glycolysis in vivo

**FIGURE 4:**
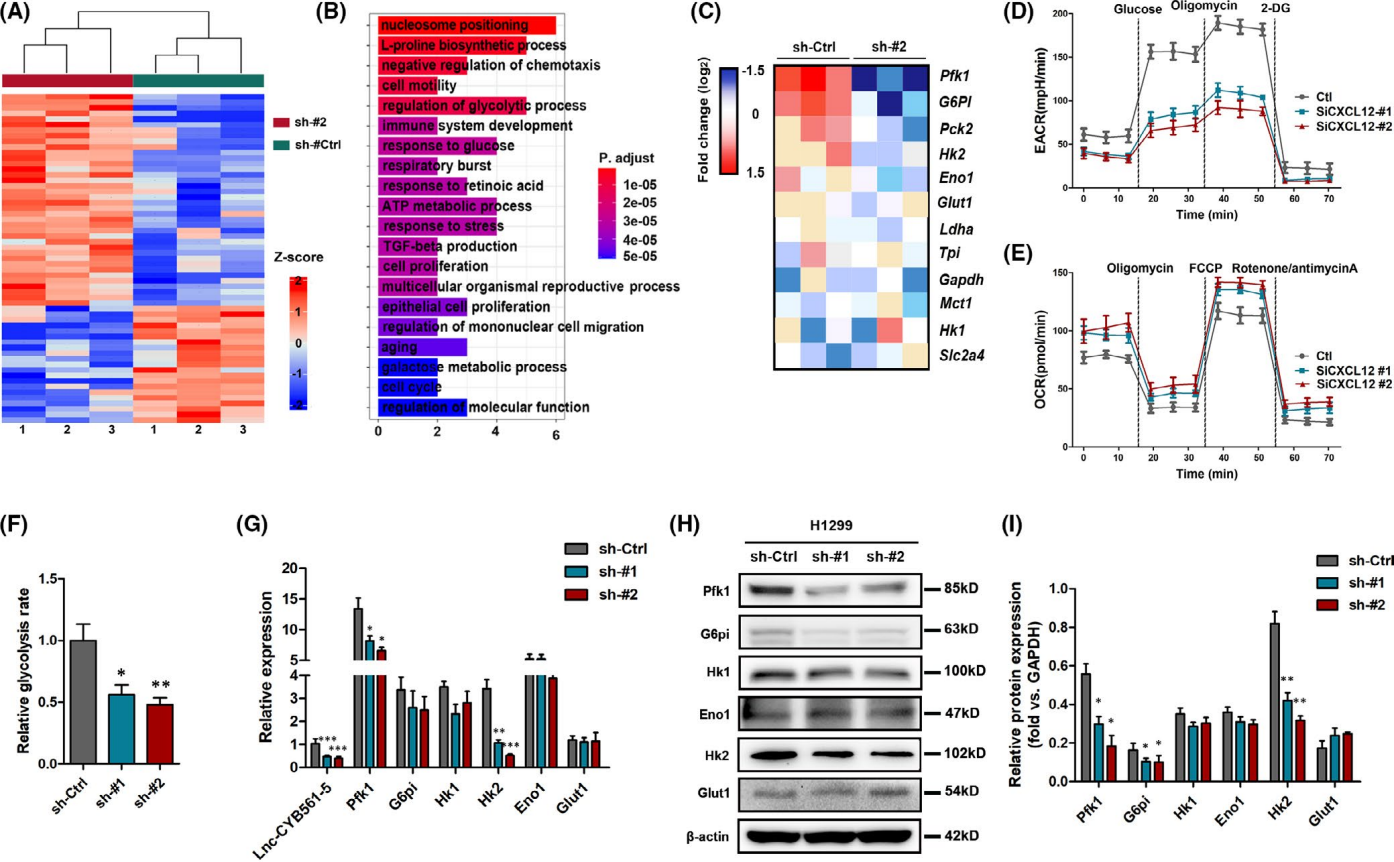
Lnc‐CYB561‐5 promotes aerobic glycolysis in vivo. (A) Cluster analysis of differentially expressed mRNAs in H1299 cells treated with the lnc‐CYB561‐5 knockdown. (B) The top 20 GO enrichment of significantly upregulated genes (fold change > 2, *p *< 0.05, top 100). (C) Heatmap depicting relative expression of known genes related to glycolysis. (D and E) Measurement of ECARO and CR in H1299 cells treated with the lnc‐CYB561‐5 knockdown. (F) Relative glycolysis rates in H1299 cells, as judged by Seahorse analyses. (G–I) Relative mRNA and protein expression levels of Pfk1, Hk1, Hk2, Eno1, G6pi and Glut1 in H1299 cells. *p < 0.05, **p < 0.01, ***p < 0.001, NS, *p *> 0.05 vs. the sh‐Ctrl group, *n* = 5. Data are presented as means ± SEM. Multiple group comparisons were performed using one‐way ANOVA followed by Tukey's post hoc test

To determine the detailed mechanism of action of lnc‐CYB561‐5 in regulating lung cancer proliferation and metastasis, we further performed RNA‐seq analysis on lnc‐CYB561‐5 knockdown treated H1299 cells (Figure 4A). GO term enrichment analysis revealed that the significantly upregulated (fold change>2, *p *< 0.05, top 50) genes were enriched for ‘nucleosome positioning’, ‘cell motility’ and ‘regulation of glycolytic process’ (Figure 4B). Heatmap analysis showed that known glycolysis‐related genes, such as pfk1, g6pi, pck2 and hk2, were upregulated after sh‐lnc‐CYB561‐5‐#2 treatment (Figure 4C). We then measured both oxygen consumption rate (OCR), an indicator of aerobic respiration, and extracellular acidification rate (ECAR), a readout of lactic acid produced from aerobic glycolysis, to determine whether the lnc‐CYB561‐5‐induced changes in metabolic phenotypes in lung cancer cells. H1299 cells with sh‐lnc‐CYB561‐5‐#2 treatment showed decreased basal and maximum EACR but increased OCR relative to the control group (Figure 4D, E). The glycolysis rates (OCR/EACR ratio) were approximately 50% lower in the control H1299 cells than in sh‐lnc‐CYB561‐5‐#2 treated H1299 cells (Figure 4F). In addition, the analysis of gene and protein expressions of Pfk1, G6pi, Hk1, Hk2, Eno1 and Glut1 were detected by qRT‐PCR and Western blotting analysis. The results indicated that Hk2 and Pfk1, which are the rate‐limiting enzymes of glycolysis, were downregulated in H1299 cells treated with sh‐lnc‐CYB561‐5‐#2 treatment (Figure 4G–I) and were upregulated in A549 cells with lnc‐CYB561‐5 overexpression (Figure S2D and Figure S2E.

### 3.5 **Identification target genes of lnc‐CYB561‐5 in NSCLC**.

**FIGURE 5:**
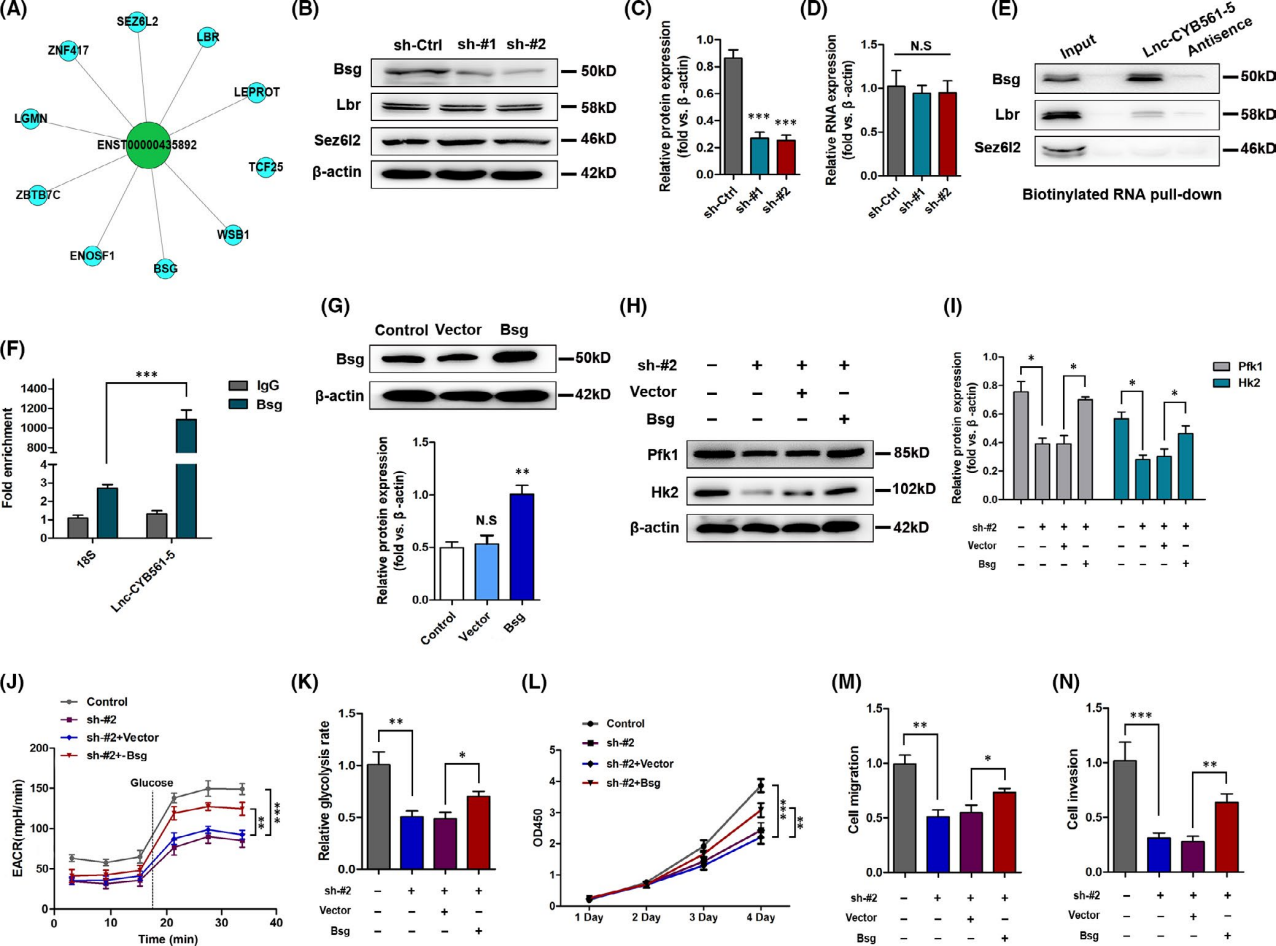
Identification target genes of lnc‐CYB561‐5 in NSCLC. (A) The co‐expression network. This network consists of lnc‐CYB561‐5 (green) and its 10 direct neighbours (blue). (B and C) Relative protein expression levels of Bsg, Lbr and Sez6l2 in H1299 cells. (D) The RNA level of Bsg in H1299 cells. ***p< 0.001, NS, *p *> 0.05 vs. the sh‐Ctrl group, *n* = 5. (E) Western blot analysis to determine the levels of Bsg, Lbr and Sez6l2 on RNA pulldown material. (F) RIP assays were performed to test the interaction of lnc‐CYB561‐5 and Bsg. Relative quantification of lnc‐CYB561‐5 and 18S rRNA in RNA‐protein complexes immunoprecipitated with IgG or Bsg from whole‐cell extracts. (G) Validation of Bsg overexpression efficiency, *p < 0.05, NS, *p *> 0.05 vs. the control group. (H and I) Western blot determination of Pfk1 and Hk2 in H1299 cells. (I) Measurement of ECAR in H1299 cells. (J) Relative glycolysis rates in H1299 cells. (K) CCK‐8 assay analysis of cell proliferation in H1299 cells. (L and M) The migration and invasion of H1299 cells. *p < 0.05, **p < 0.01, ***p < 0.001 vs. the indicated group, *n* = 5. Data are presented as means ± SEM. Multiple group comparisons were performed using one‐way ANOVA followed by Tukey's post hoc test

We identified ten potential target genes of lnc‐CYB561‐5 by examining the regulation of cell proliferation and metastasis by lnc‐CYB561‐5 in H1299 cells by determining the correlation between the expression level of lnc‐CYB561‐5 and corresponding protein‐coding genes using co‐expression network analysis (Figure 5A). Western blot and qRT‐PCR analysis showed that lnc‐CYB561‐5 knockdown could reduce the expression level of basigin (Bsg), but had no effect on the expressions of delta‐sterol reductase (Lbr) and seizure 6‐like protein 2 (Sez6l2), which have been associated with lung cancer as previously reported (Figure 5B–D, Figure S3). Further Western blot analysis on RNA pulldown material revealed that lnc‐CYB561‐5 was associated with the expression of Bsg but not Lbr or Sez6l2 (Figure 5E). Moreover, the results of RIP assays confirmed the significant interaction of lnc‐CYB561‐5 with Bsg in H1299 cells (Figure 5F). In addition, Bsg overexpression significantly reversed the downregulation of the expressions of Hk2 and Pfk1, as well as the lnc‐CYB561‐5 knockdown‐induced ECAR, and glycolysis rate (Figure 5G–J). From a functional point of view, Bsg overexpression also effectively induced the cell proliferation, migration and invasion in H1299 cells treated with sh‐lnc‐CYB561‐5‐#2 treatment (Figure 5K–M).

## 4 DISCUSSION

Currently, lung cancer is still the leading cause of cancer‐related deaths worldwide. Recently, the crucial role of lncRNAs in lung cancer development has been extensively investigated. However, although many researchers believe that lncRNAs have great potential as biomarkers or therapeutic targets for lung cancer, but few have been experimentally validated and functionally annotated.^24^, ^25^, ^26^ In this study, we found that lnc‐CYB561‐5 was highly expressed in NSCLC and showed a close correlation with the proliferation, metastasis and metabolic reprogramming of lung cancer cells, suggesting that it might represent an independent prognostic biomarker in NSCLC.

Although most lncRNAs lack protein‐coding ability, they are closely involved in cell proliferation, differentiation and cell metabolism, and other biological processes, especially those involved in cancer pathogenesis.^9^, ^27^, ^28^ Lnc‐CYB561‐5, a novel transcript antisense to TANC2 located in chromosome 17, is 491 bp in length. At present, little is known about the biological activity, molecular function and role of lnc‐CYB561‐5 in tumorigenesis. Tang et al.^29^ used the R software to screen for differentially expressed genes in lung adenocarcinoma from the TCGA database and found that lnc‐CYB561‐5 was upregulated in NSCLC tissues and was associated with the prognosis of NSCLC. However, no experimental studies or clinical data have shown that lnc‐CYB561‐5 is an important regulator or biomarker of cancer progression. In this study, we performed RNA‐seq and qRT‐PCR analysis and the result demonstrated that lnc‐CYB561‐5 was highly expressed in NSCLC tissue and lung cancer cell lines. Our in vivo and in vitro results demonstrated that knockdown of lnc‐CYB561‐5 caused a decrease in cancer cell proliferation and invasion. These findings suggested that lnc‐CYB561‐5 may serve as a potential therapeutic target for NSCLC.

Feng et al. proposed that metabolic reprogramming represents an important survival mechanism in cancer cells and is currently recognized as a hallmark of cancer.^30^, ^31^, ^32^ To facilitate sustained cell survival, excessive proliferation, metastasis and escape from the immune system, cancer cells would actively choose glycolysis as the main way to obtain energy, even in an oxygen‐rich environment, a process known as the Warburg effect.^33^ Besides providing ATP, aerobic glycolysis generates less reactive oxygen species and more antioxidants, lipids and nonessential amino acids which can promote increased survival of cancer cells.^34^ Increasing evidence supports the view that some lncRNAs can serve as ‘metabolic regulators’ by participating in cellular energy programming. Wang and co‐workers proposed that lnc‐LINRIS could promote aerobic glycolysis in colorectal cancer by stabilizing IGF2BP2.^35^ Another study by Wang et al.^36^ also provided compelling evidence that lnc‐HULC directly binds to lactate dehydrogenase A (LDHA) and pyruvate kinase M2 (PKM2), leading to the phosphorylation of these two enzymes, thereby promoting glycolysis. In this study, upon knockdown of lnc‐CYB561‐5, gene enrichment analysis identified a significant decrease in metabolic pathways including energy metabolism and glycolytic process genes upon lnc‐CYB561‐5 knockdown, such as Pfk1, Hk2 and G6pi. Extracellular flux measurements indicated that the downregulation of lnc‐CYB561‐5 inhibited aerobic glycolysis. Together, these results suggest that lnc‐CYB561‐5 is an important metabolic regulator in the context of NSCLC.

Mechanistically, interacting with proteins is a crucial way for lncRNAs to exert their biological effects. In this study, we predicted that Bsg may be an interacting protein of lnc‐CYB561‐5 and further confirmed this prediction by RNA pulldown and RIP assays in vitro. Bsg, also known as CD147, is expressed in a variety of cell types and is involved in the processes of organ development, wound healing, inflammation and tumour progression.^37^, ^38^ Recent studies show that Bsg is highly expressed in several cancer cell types and is associated with tumour size, tumour stage, progression and prognosis.^39^, ^40^, ^41^ Zhou et al.^42^ determined that Bsg recycling is required for lung cancer cell migration and invasion, and targeting Bsg recycling may be a rational strategy for lung cancer therapy. Albrechtsen et al.^43^ also found that Bsg is proteolytically shed from the cell surface and high concentrations of soluble Bsg in the blood indicates poor prognosis in cancer patients. Our results showed that knockdown of lnc‐CYB561‐5 led to decreased expression of Bsg in H1299 cells. Overexpression of Bsg can effectively block the downregulation of the glycolysis level, as well as the decrease of cell proliferation, migration and invasion activity caused by lnc‐CYB561‐5 knockdown in H1299 cells. Therefore, the involvement of lnc‐CYB561‐5 in the regulation of lung cancer cell metabolism, proliferation and metastasis is Bsg dependent.

Despite the significance of the findings in this study, there are several limitations that warrant discussion. Firstly, although our evidence supports the conclusion that lnc‐CYB561‐5 promotes the metastasis of lung cancer cells, further experimental metastasis assay, such as in vivo bioluminescence imaging of mice, is needed to be performed to confirm its role in lung cancer metastasis. Secondly, increased analysis of the function of Bsg on cell proliferation, migration and invasion is necessary to further prove that BSG is the target of lnc‐CYB561‐5.

In conclusion, we found that lnc‐CYB561‐5 can promote NSCLC cell proliferation and metastasis in vitro and in vivo. Lnc‐CYB561‐5 interacted with Bsg to promote the expression of HK2 and Pfk1 and further led to aerobic glycolysis in NSCLC. These data provided important insights into the use of lncRNA as a biomarker or therapeutic target for NSCLC.

## CONFLICT OF INTEREST

The authors declare that they have no competing interests.

## AUTHOR CONTRIBUTIONS

**Longfei Li:** Conceptualization (equal); Data curation (lead). **Zhimin Li:** Methodology (equal). **Jingming Qu:** Investigation (equal); Methodology (equal). **Xiangju Wei:** Software (equal). **Feng Suo:** Data curation (equal); Investigation (equal). **Jilei Xu:** Formal analysis (equal); Methodology (equal). **Chang Chen:** Investigation (equal); Project administration (equal). **Shiying Zheng:** Conceptualization (lead); Funding acquisition (lead). **Xiucheng Liu:** Data curation (equal); Writing‐review & editing (equal).

## Supporting information

Supplementary Material

## Data Availability

All relevant data have been presented in the manuscript. All requests for or questions about the data can be initiated by contacting 2010692@tongji.edu.cn.
