# Supplementary material for: Novel long non‐coding RNA CYB561‐5 promotes aerobic glycolysis and tumorigenesis by interacting with basigin in non‐small cell lung cancer
Source: J Cell Mol Med. 2022 Jan 22;26(5):1402–12. doi: 10.1111/jcmm.17057 (PMC8899181; doi:10.1111/jcmm.17057)
Supplement: Supplementary file 1 — Supplementary Material [file JCMM-26-1402-s001.docx]

**Supplementary Meterial**

**Supplementary Table 1: Primer sequences used in this study**

| **Gene** | **Forward (5’-3’)** | **Reverse (5’-3’)** |
| --- | --- | --- |
| Lnc-CYB561-5 | TCTTCAGGCCTCTCTTCGAG | CCTGGAACAAAACCCACAGT |
| HK1 | GCAGGGACCACACTTTGAGAACC | AGAGGAGGAAGAGGACAGCACAC |
| HK2 | GTGAACGATGCTCCTGCTCTGAAG | CTCCTCAACGGCAGCCACAATG |
| PFK1 | CAACCGATTCCGCTCCAACTTCC | GTACCACCTTCACTTAACCAACCTCTG |
| ENO1 | AAAGCTGGTGCCGTTGAGAA | GGTTGTGGTAAACCTCTGCTC |
| G6PI | AACCGCTCCAACACTCCCATTAAC | TCGCCACTACGCACTTTATGACAG |
| GLUT1 | GAGGTAGTCAGACGGAAGCTA | GAGGGGAACATGCAGTCATTT |
| BSG | GAAGTCGTCAGAACACATCAACG | TTCCGGCGCTTCTCGTAGA |
| β-actin | CTCCATCCTGGCCTCGCTGT | GCTGTCACCTTCACCGTTCC |

**Supplementary Table 2. The clinic-pathological factors of NSCLC patients**

| Variables | n | Lnc-CYB561-5 expression | | *P* value |
| --- | --- | --- | --- | --- |
|  |  | Low (n=29) | High (n=45) |  |
| Gender |  |  |  | 0.483 |
| Male | 42 | 18 | 24 |  |
| Female | 32 | 11 | 21 |  |
| Age |  |  |  | 1.000 |
| ≤50 | 56 | 22 | 34 |  |
| ＞50 | 18 | 7 | 11 |  |
| Histological classification |  |  |  | 1.000 |
| LUSC | 24 | 9 | 15 |  |
| LUAD | 50 | 20 | 30 |  |
| Tumor stage |  |  |  | 0.002 |
| I~II | 31 | 19 | 12 |  |
| III~IV | 43 | 10 | 33 |  |
| Lymph node metastasis |  |  |  | 0.004 |
| Positive | 28 | 5 | 23 |  |
| Negative | 46 | 24 | 22 |  |
| EGFR status |  |  |  | 0.764 |
| Positive | 14 | 5 | 9 |  |
| Negative | 26 | 13 | 13 |  |

*LUSC, lung squamous carcinoma; LUAD, lung adenocarcinoma; EGFR, Epidermal growth factor receptor. Lnc-CYB561-5 expression greater than the average was considered to be high, and lnc-CYB561-5 expression less than the average was considered to be low.

**Supplementary Figures**

**Supplementary Figure 1. The correlation of lnc-CYB561-5 with tumor pathological classification and tumor stage.**  (A) Lnc-CYB561-5 was up-regulated in both LUAD and LUSC tissues. (B, C) The relationship between lnc-CYB561-5 expression and tumor stage according to TCGA database.

**
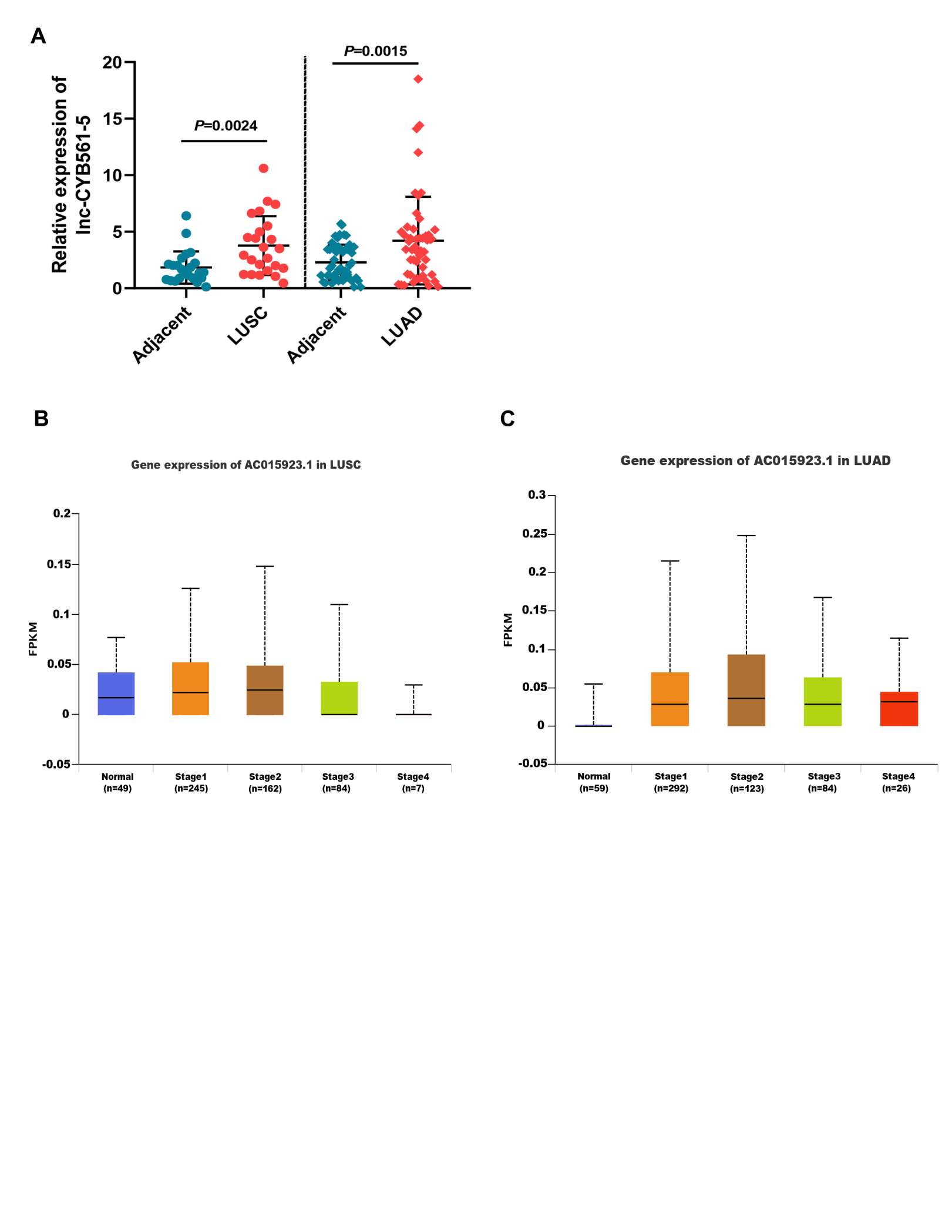
**

**Supplementary Figure 2. The effect of lnc-CYB561-5 on the proliferation, migration, invasion and glycolysis in A549 cells.**

(A) CCK-8 assay analysis of cell proliferation in A549 cells. (B) The expression levels of metastasis associated genes of NSCLC in A549 cells. (C) The migration and invasion of A549 cells with lnc-CYB561-5 over-expression treatment, bar=50 μm. (D, E) Western blot and RT-PCR for the levels of Pfk1 and Hk2 in A549 cells, **P*＜0.05;***P*＜0.01 vs the vector group, n=3.


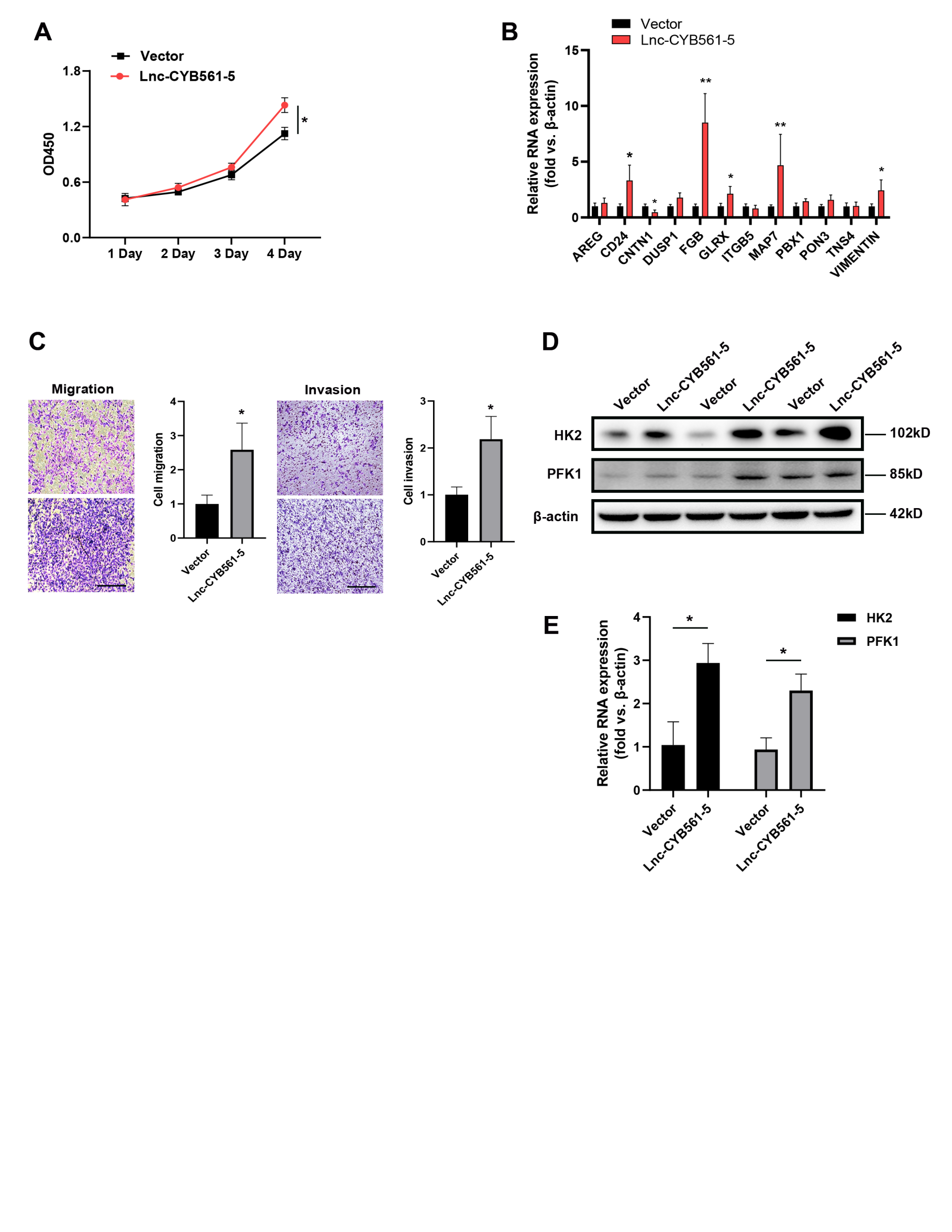


**Supplementary Figure 3. The expressions of ten potential target mRNA of lnc-CYB561-5 in lung cancer according to**

**TCGA database.** The expressions of WSB1, TCF25, LEPROT, LBR, SEZ6L2, ZNF417, LGMN, ZBTB7C, ENOSF1 and BSG

in NSCLC and normal tissues according to TCGA database.

**
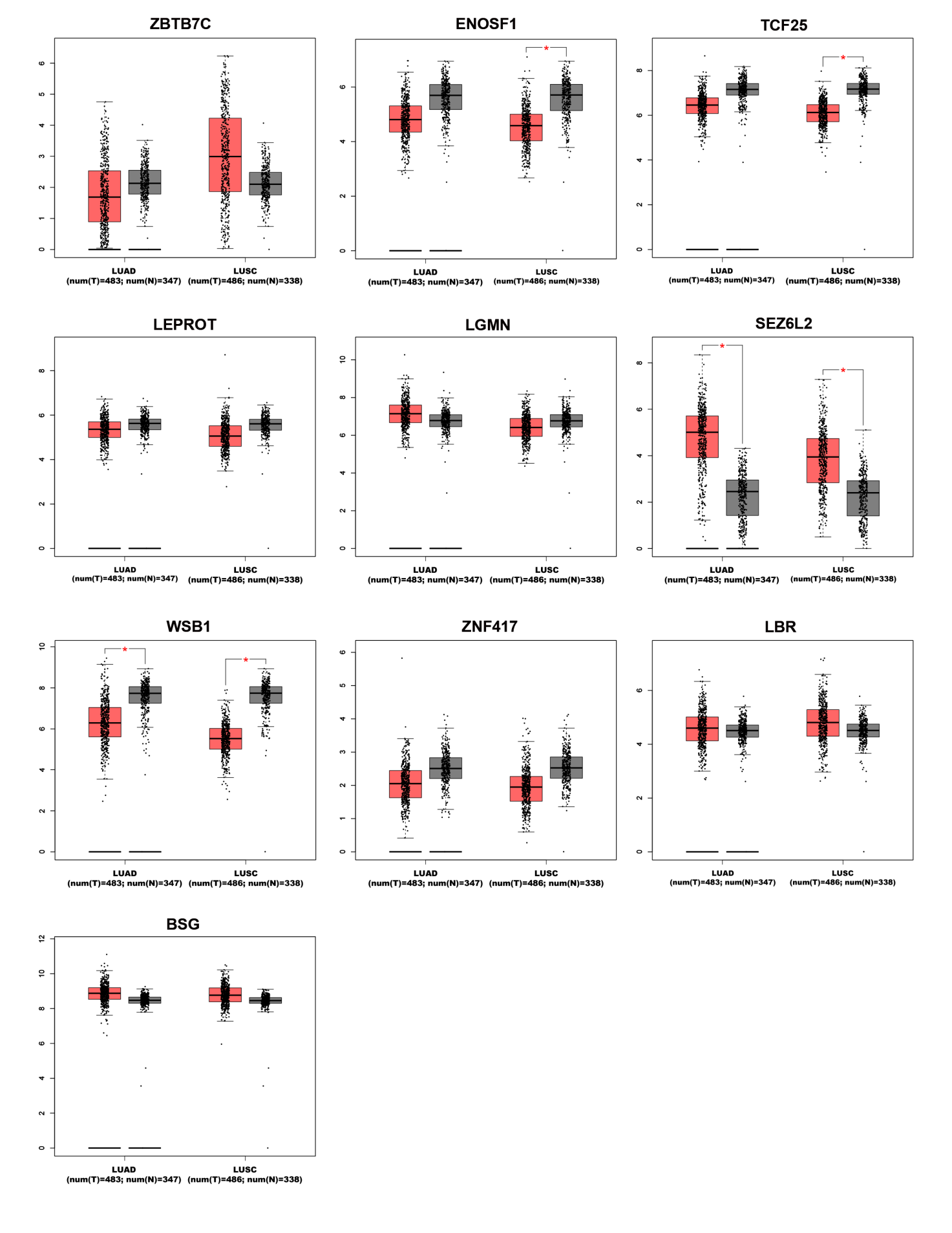
**
